# Supplementary material for: Bioinspired adaptable multiplanar mechano-vibrotactile haptic system
Source: Nat Commun. 2024 Sep 11;15:7631. doi: 10.1038/s41467-024-51779-8 (PMC11390908; doi:10.1038/s41467-024-51779-8)
Supplement: Supplementary file 1 — Supplementary Information [file 41467_2024_51779_MOESM1_ESM.pdf]

Supplementary Information: Bioinspired adaptable multiplanar mechano-vibrotactile haptic system

Sara-Adela Abad[1, 2*], Nicolas Herzig[3], Duncan Raitt[1], Martin Koltzenburg[4], and Helge Wurdemann[1]

[1]Department of Mechanical Engineering, University College London, London, WC1E 7JE, UK
[2]Faculty of Agriculture and Renewable Natural Resources, Universidad Nacional de Loja, Loja, 110101, Ecuador.
[3]School of Engineering and Informatics, University of Sussex, Brighton, BN1 9RH, UK
[4]Queen Square Institute of Neurology, University College London, London, WC1N 3BG, UK

This PDF file contains:

- Supplementary Figure 1: Fingertip interface: manufacturing process.
- Supplementary Figure 2: Raw force signal delivered by the BAMH system.
- Supplementary Figure 3: Raw force data of the BAMH system in the time and frequency domain for pulse stimulus, with $\delta = 75\,\%$, between 0 Hz and 50 Hz.
- Supplementary Figure 4: Raw force data of the BAMH system in the time and frequency domain for pulse stimulus, with $\delta = 75\,\%$, between 55 Hz and 100 Hz.
- Supplementary Figure 5: Raw force data of the BAMH system in the time and frequency domain for pulse stimulus, with $\delta = 75\,\%$, between 105 Hz and 130 Hz.
- Supplementary Figure 6: Participants' stimuli classification: accuracy and precision.
- Supplementary Figure 7: Distal phalanx two-point stimuli classification performance using the haptic system.
- Supplementary Figure 8: Trials where participants did not feel the stimuli.
- Supplementary Figure 9: Fingers dimensions.
- Supplementary Figure 10: Manufacturing process and experimental setup for the characterisation of the flat sample.
- Supplementary Figure 11: Index finger stimuli differentiation stimuli pairs illustration.
- Supplementary Table 1 : Deviation of force data over time and flat samples.
- Supplementary Table 2 : Average force at each frequency across duty cycles and pipe length.

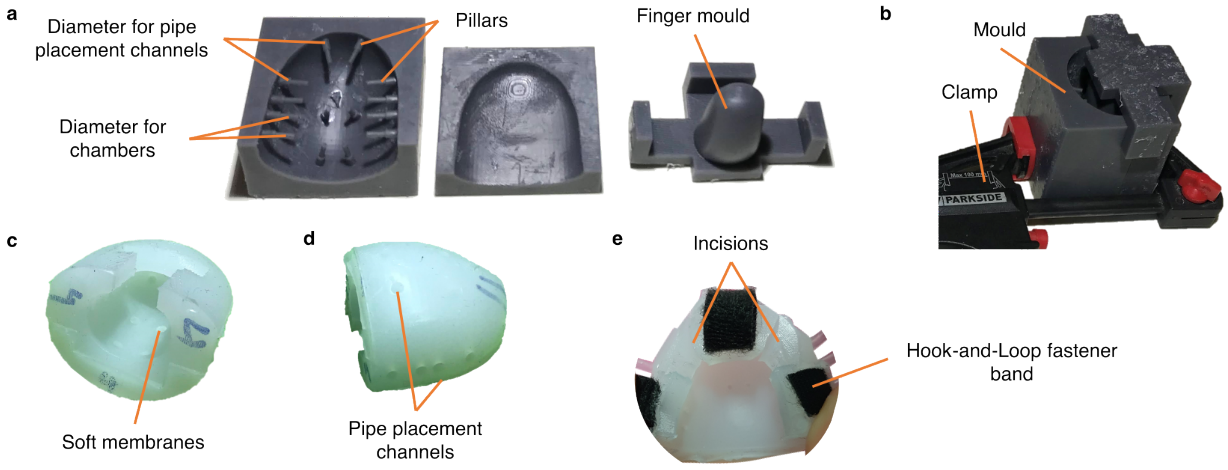

Supplementary Figure 1 : **Fingertip interface: manufacturing process.** **a** Parts of the mould used to obtain the finger-shaped soft interface with pillars to form the chambers during curing. **b** Mould ready for pouring the Dragon Skin 20. Soft sleeve with the **c** soft membranes, **d** pipe placement channels obtained after removing from mould and **e** the incisions and fastener bands.

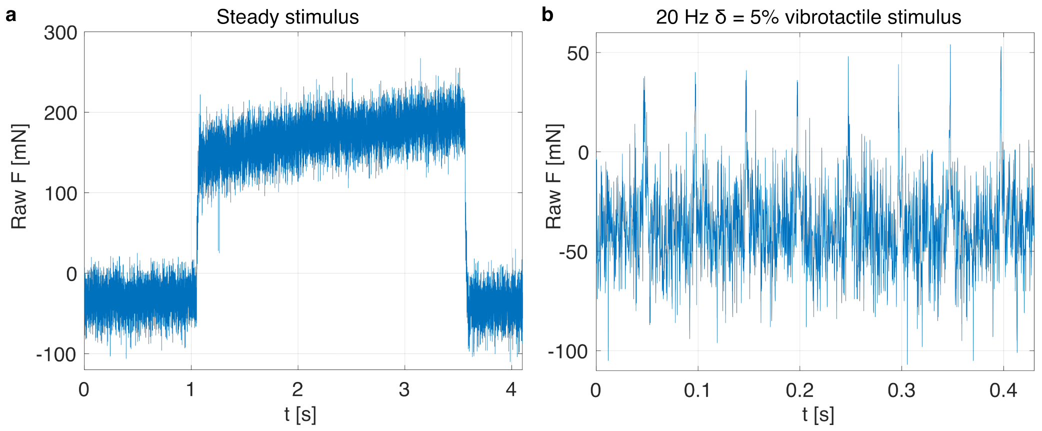

Supplementary Figure 2 : **Raw force signal delivered by the BAMH system.** **a** this steady signal is representative as the stimulus intensity changes across the sensitivity test until the intensity threshold is determined, and **b** part of the vibro-tactile stimulus (20 Hz 5 % duty cycle) used for the differentiation test.

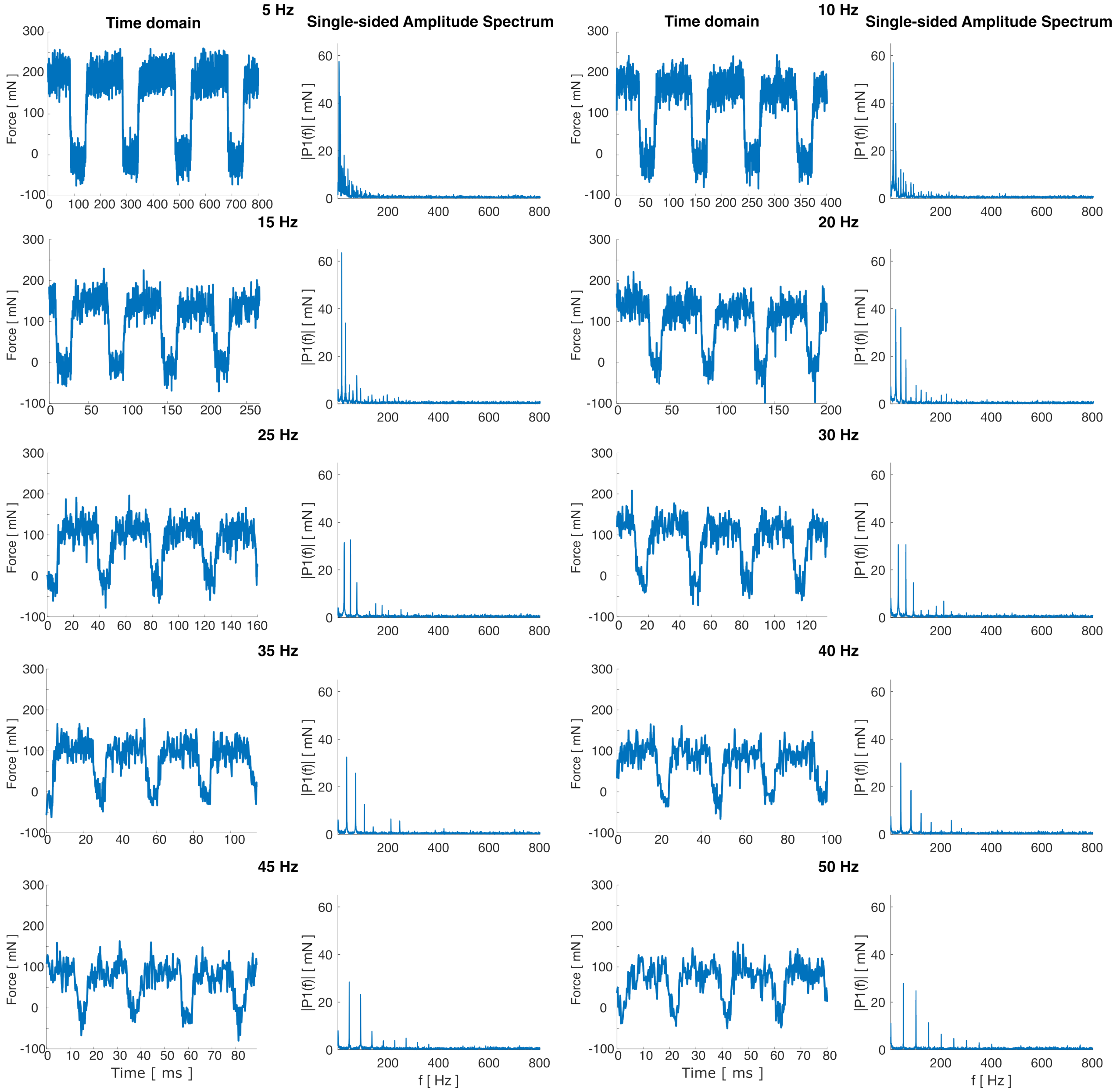

Supplementary Figure 3 : **Raw force data of the BAMH system in the time and frequency domain for pulse stimulus, with $\delta = 75\,\%$, between $0\,\mathrm{Hz}$ and $50\,\mathrm{Hz}$.** The frequency domain plots demonstrate the BAMH system's capability to deliver pulse stimulus by exhibiting the spectral coefficients at the frequencies that are a multiple of the stimulus frequency.

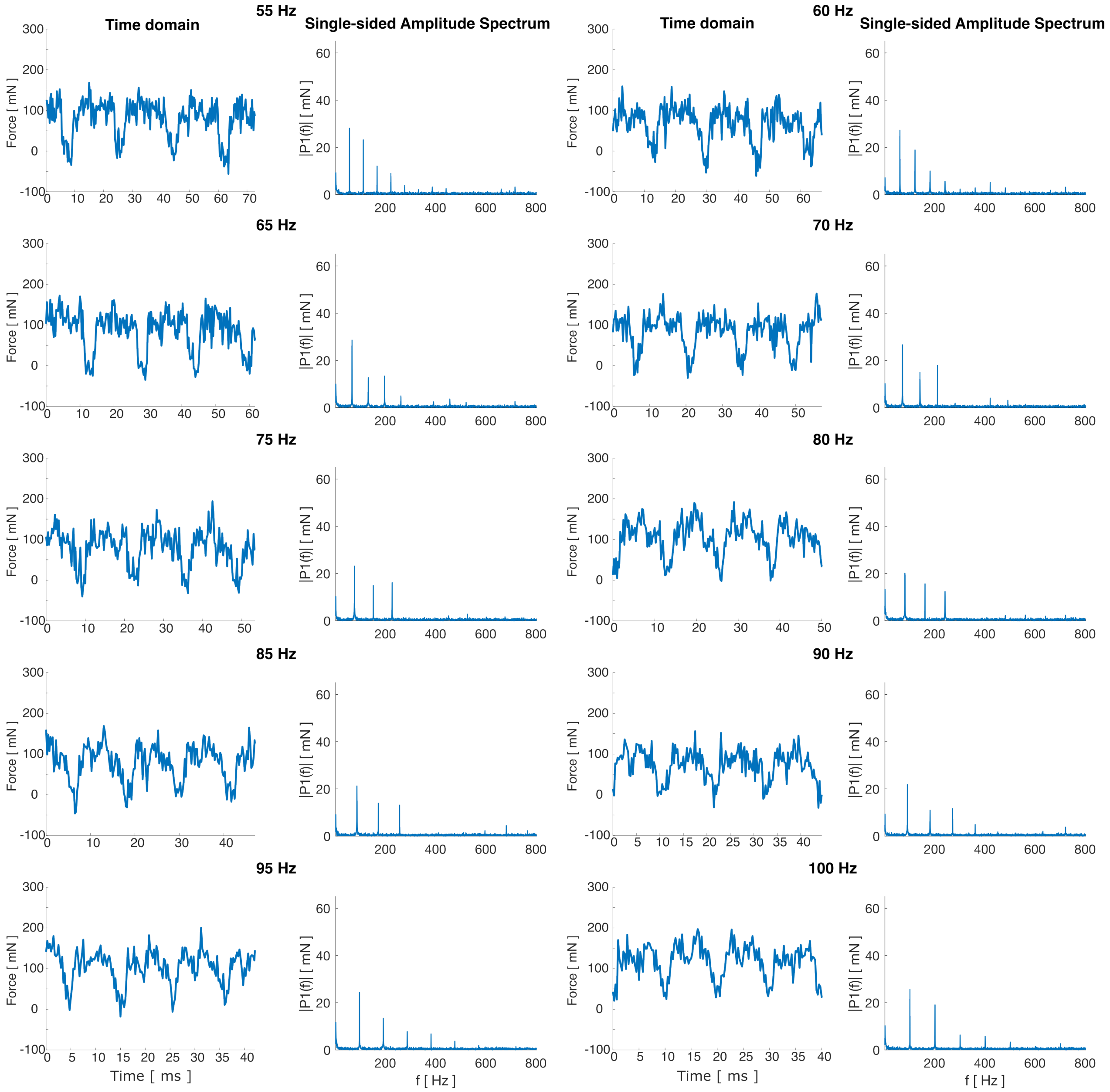

Supplementary Figure 4 : **Raw force data of the BAMH system in the time and frequency domain for pulse stimulus, with $\delta = 75\,\%$, between $55\,\mathrm{Hz}$ and $100\,\mathrm{Hz}$.** The frequency domain plots demonstrate the BAMH system's capability to deliver pulse stimulus by exhibiting the spectral coefficients at the frequencies that are a multiple of the stimulus frequency.

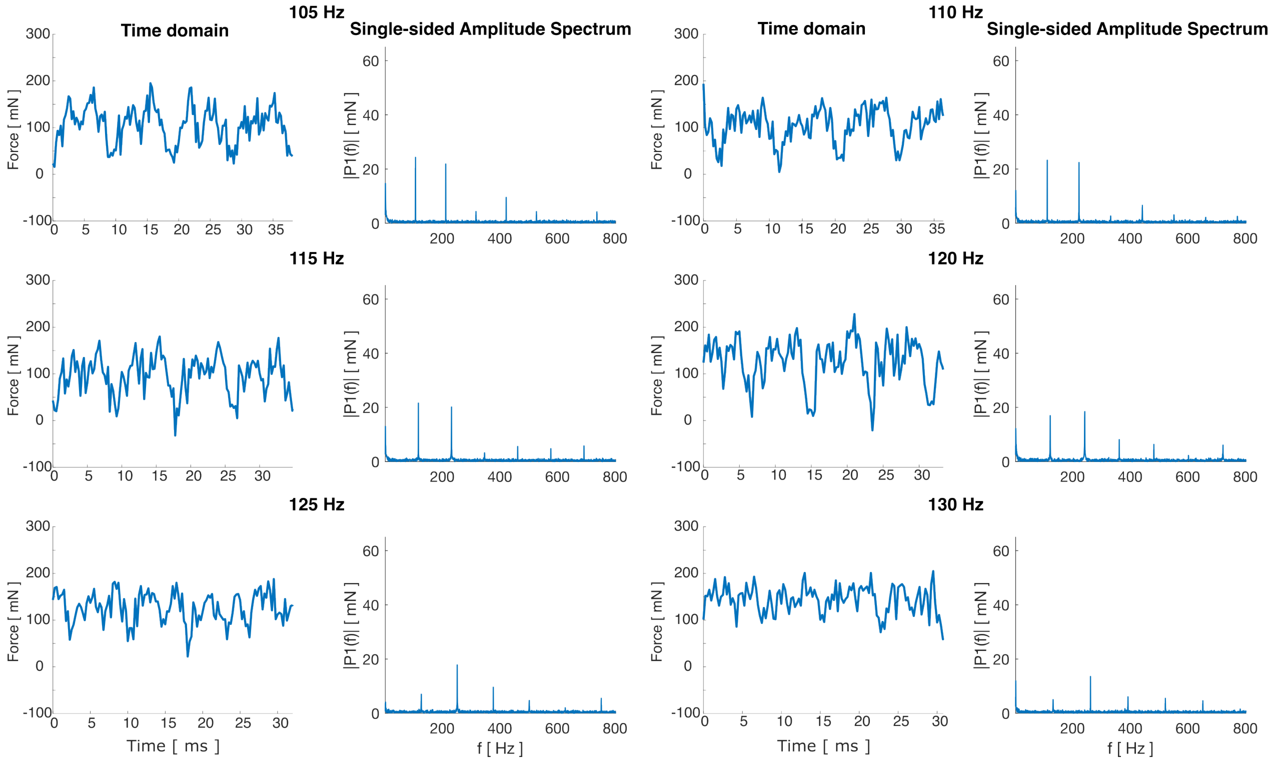

Supplementary Figure 5 : **Raw force data of the BAMH system in the time and frequency domain for pulse stimulus, with $\delta = 75\,\%$, between 105 Hz and 130 Hz.** The frequency domain plots demonstrate the BAMH system's capability to deliver pulse stimulus by exhibiting the spectral coefficients at the frequencies that are a multiple of the stimulus frequency..

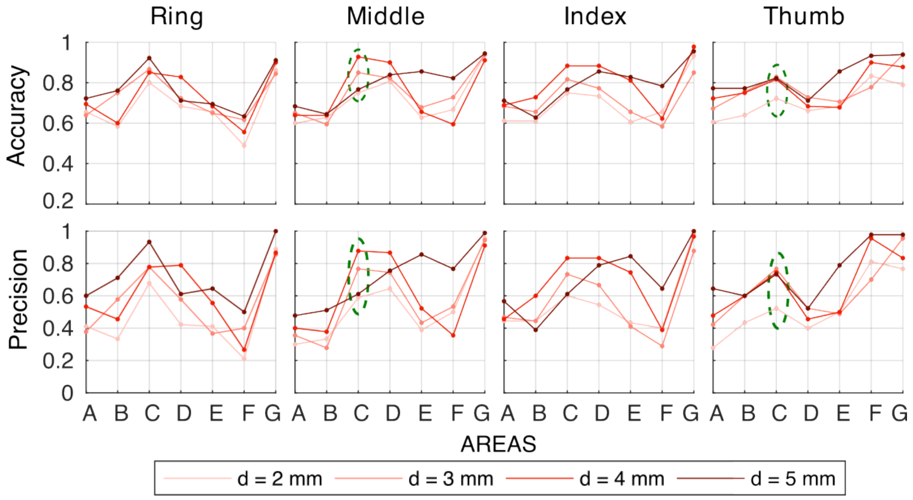

Supplementary Figure 6 : **Participants' stimuli classification: accuracy and precision**. The distance between the stimuli is represented by the darkness of the line (e.g., the darkest and lightest colored lines correspond to the 5 mm and 2 mm distance, respectively). The green dashed ellipses highlight that a greater distance between the stimuli does not necessarily imply the highest accuracy and precision.

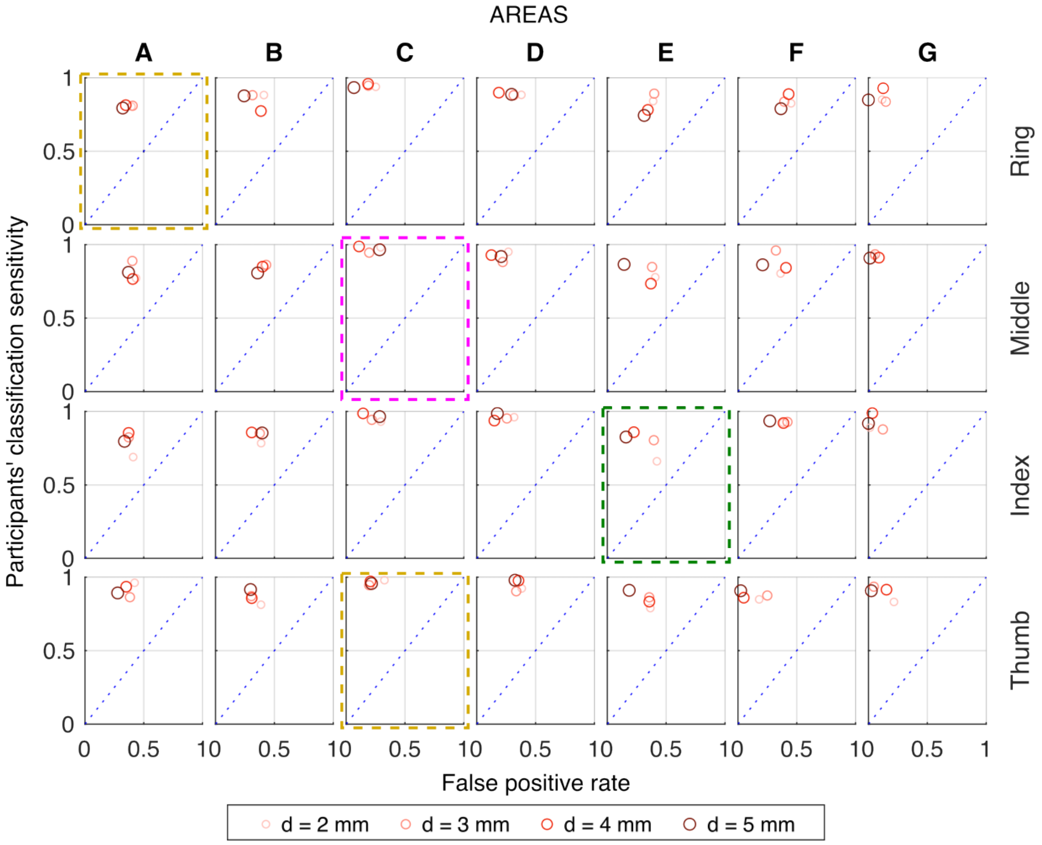

Supplementary Figure 7 : **Distal phalanx two-point stimuli classification performance using the haptic system**. The distance is highlighted by the circle diameter and the darkness of its line. For instance, 5 mm is represented by the circle with the largest diameter and the coloured line that is the darkest red. The best possible classification outcome is at the top left corner (coordinate (0,1)), and a random guess is along the diagonal. The green rectangle exhibits the closest to random classifier for area E of the index finger for $d = 4$ mm. The fuchsia rectangle demonstrates that participants can better differentiate with a shorter distance between stimuli, depending on the finger and area. The yellow rectangle illustrates cases where the results are similar across distances.

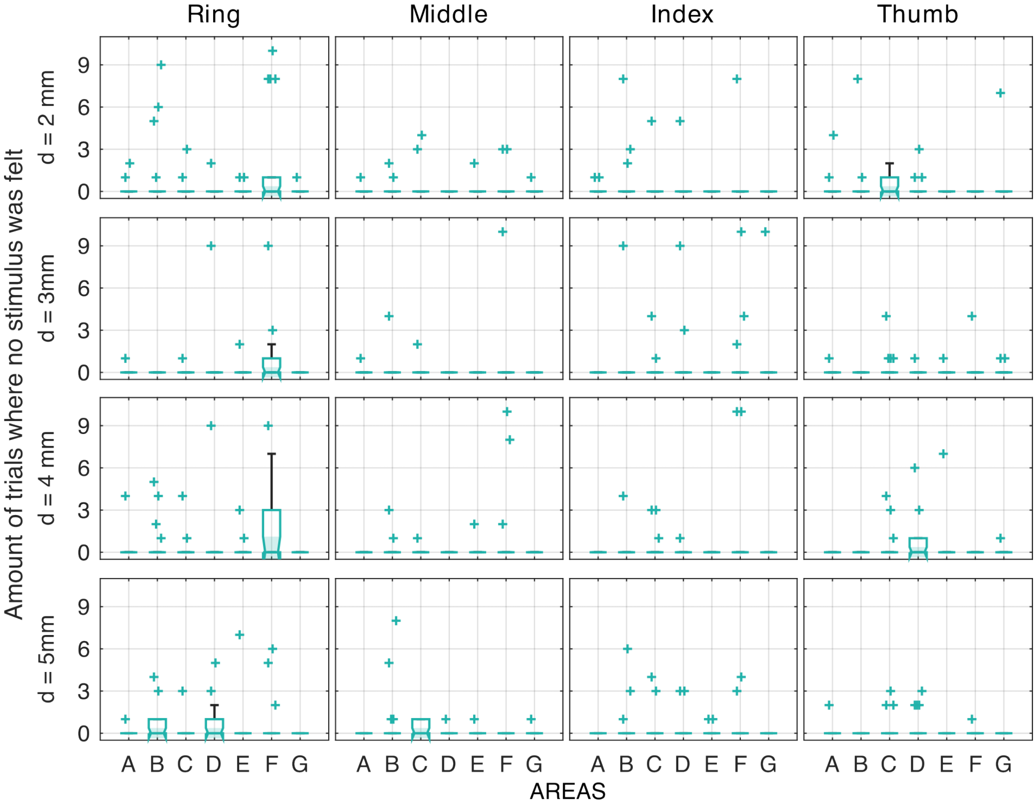

Supplementary Figure 8 : **Trials where participants did not feel the stimuli**. The median value is highlighted with the box notch while the box limits are the second and third quartiles. The outliers are represented with +.

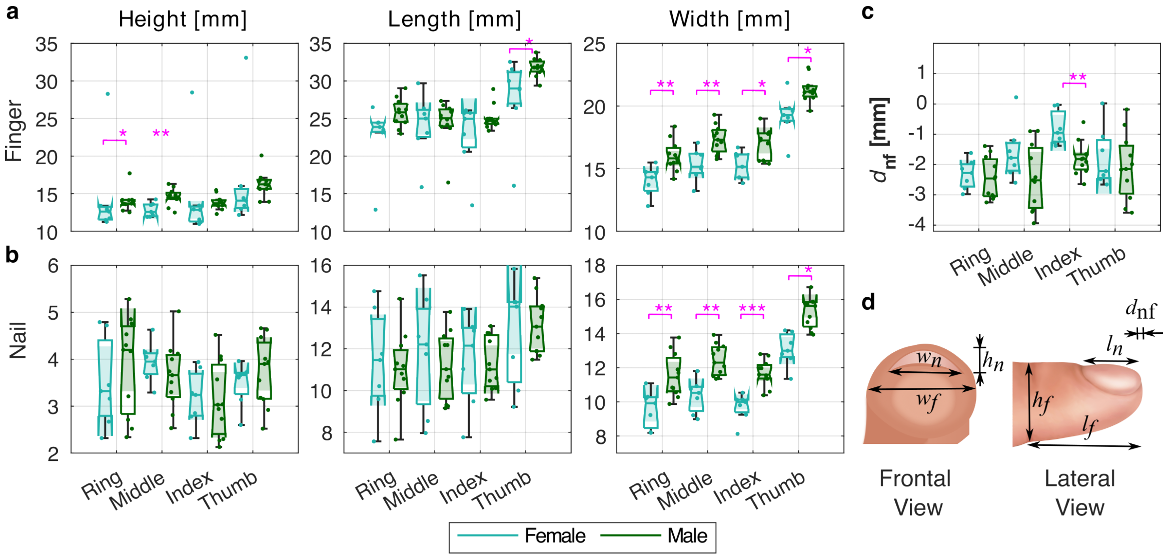

Supplementary Figure 9 : **Fingers dimensions**. **a** Finger height, length, and width. **b** Nail height, length, and width. **c** Distance between the tip of the finger and the nail, d_{nf}. The notch represents the data points and the median. The second and third quartiles, represented by a box, are generally in a similar range across females and males. The exceptions are highlighted with $*$, $**$, and $***$, which represent statistical significance with $p \leq 0.05$, $p \leq 0.01$, and $p \leq 0.001$, respectively. **d** Illustration of the dimensions.

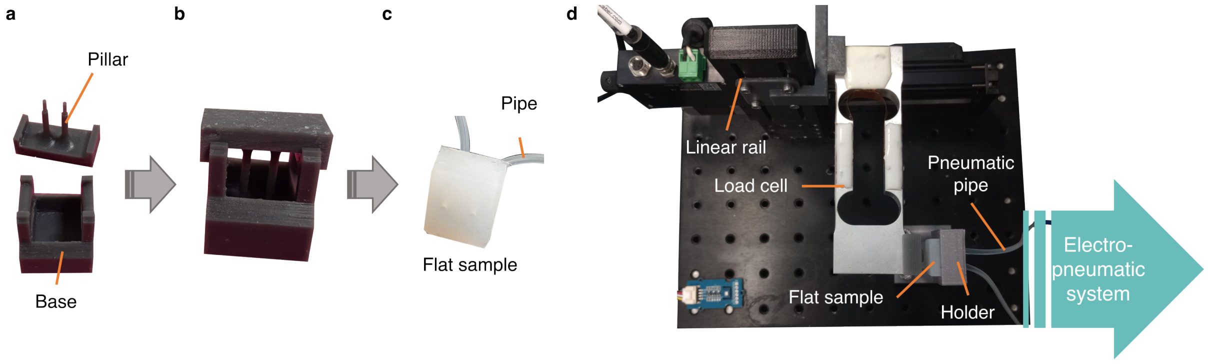

Supplementary Figure 10 : **Manufacturing process and experimental setup for the characterisation of the flat sample.** **a** Parts and **b** fully assembled mould to manufacture the **c** flat sample of the fingertip interface. **d** Experimental setup used for the characterisation.

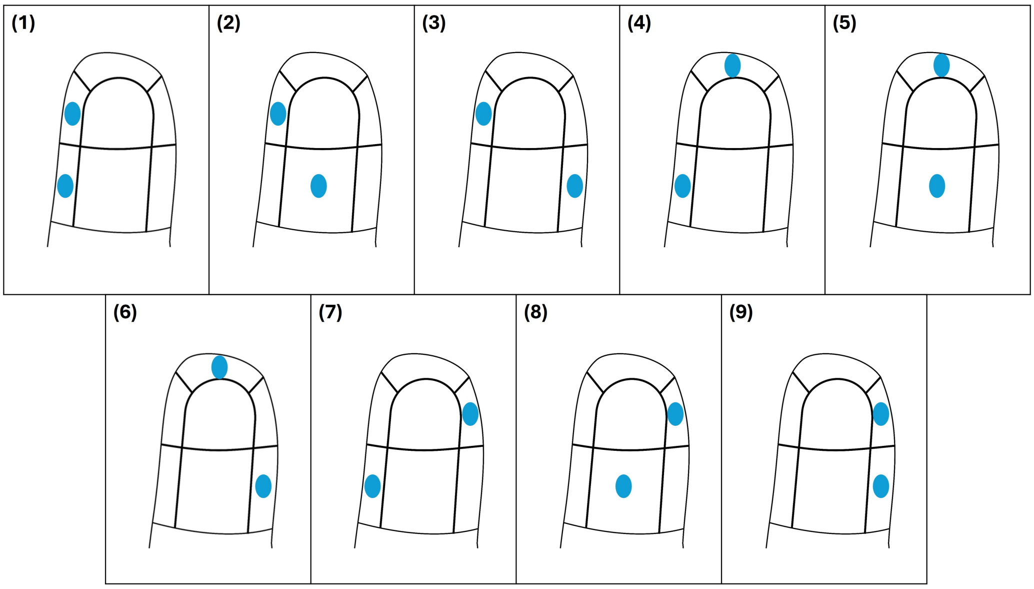

Supplementary Figure 11 : **Index finger stimuli differentiation stimuli pairs illustration.** Participants were handed this illustration that presents, from the top view, the evaluated nine-pair combinations. The top view was employed to facilitate the association of the received stimulus and those from the illustration. The vibrotactile pulse stimuli characteristics are the same used for the distal phalanx stimuli differentiation (internal pressure of 154.8 kPa, $f = 20\,\text{Hz}$, $\delta = 5\,\%$, and simultaneous stimuli application during 1.5 s). The paired areas are: **(1)** DE, **(2)** DF, **(3)** DA, **(4)** CE, **(5)** CF, **(6)** CA, **(7)** BE, **(8)** BF, and **(9)** BA.

Supplementary Table 1 : **Deviation of force data over time and flat samples.**

Testing data	Trained 2nd order polynomial fit	Root Mean Square Deviation - RMSD			
		Duty cycle			
		100 %	5 %		
		Pipe length			
		60 mm	60 mm	150 mm	250 mm
Base	Base	11.07mN	5.64mN	3.96mN	4.13mN
Week 1	Week 1	11.99 mN	5.59 mN	4.90 mN	3.84 mN
Week 1	Base	11.84 mN	5.72 mN	5.45 mN	4.13 mN
Week 2	Week 2	11.09 mN	5.84 mN	5.01 mN	4.40 mN
Week 2	Base	11.58 mN	6.23 mN	7.20 mN	6.57 mN
Flat sample 2	Flat sample 2	6.51 mN	4.60 mN	3.20 mN	2.13 mN
Flat sample 2	Base	21.74 mN	6.46 mN	2.31 mN	5.89 mN

i refers to s for steady stimulus and v for vibrotactile pulse stimulus, respectively.

RMSDs, between testing data and 2nd-order polynomial fits produced from training data, are used to track how chamber output varies across time and two different flat samples.

Supplementary Table 2 : **Average force at each frequency across duty cycles and pipe length.**

Duty cycle	Pipe length	Average force in mN 20 Hz	25 Hz	40 Hz	55 Hz	60 Hz	80 Hz	90 Hz	95 Hz	100 Hz	120 Hz	130 Hz	135 Hz
5 %	60 mm	45.3	29.6	10.8	10.6	10	10	10	9.9	10	9.5	9.1	8.8
	150 mm	28.8	20.8	10.6	10.2	9.5	9.3	9.5	9.5	9.7	8.9	8.9	8.5
	250 mm	20.8	15.4	10.8	10.5	11.1	9.5	10.2	9.8	9.8	9.1	9.2	8.9
10 %	60 mm	105.3	74	57.7	25.9	17.6	11.7	10.6	9.8	9.7	8.9	8.7	8.8
	150 mm	61.5	43.7	40	21.8	17.1	12.5	12	12.1	10.7	9	8.7	8.6
	250 mm	34.5	28.5	30.7	16.4	14.1	11.1	10	10	9.6	9.4	9.2	9.3
25%	60 mm	115.4	91.9	60.7	32.8	25.5	41.6	18	15.5	14.7	13.5	12.4	12.7
	150 mm	46	41.6	40.5	27.2	20.5	39.5	19.7	17.2	16	13.2	11.9	12.4
	250 mm	29.7	26.9	31.5	25.4	20	28.7	15	14.4	14.2	12.7	11.8	11.9
50%	60 mm	99.8	76.6	45.8	42	30.4	39.7	18.5	16	15.5	14	14.7	15.1
	150 mm	45.9	35.9	25	27.2	23.3	50	24.3	19.7	17.3	14.7	13	14
	250 mm	30.8	28.6	26.7	32.4	27.1	30.7	17.3	16.4	15.6	13.7	12.9	13.1
75%	60 mm	96.8	78.9	70.9	44.2	38.1	58.5	34.9	33.5	34.4	34.9	88.3	140
	150 mm	47.9	46.9	54.7	40.1	33.4	66.6	43.1	39.5	37.7	32.1	73.3	132.5
	250 mm	42.1	41.7	50.6	42.2	34.5	47	32	31.7	34.2	31.7	56.3	115.6

Usable frequencies are in the range from 16 mN to 105.4 mN. This range is defined by double the residual force and the highest low-frequency force (also denoted as saturation).

The full table, with frequency steps of 5 Hz, is available at the University College London Database DOI:10.5522/04/26169838.
