# Supplementary material for: Bioinspired adaptable multiplanar mechano-vibrotactile haptic system
Source: Nat Commun. 2024 Sep 11;15:7631. doi: 10.1038/s41467-024-51779-8 (PMC11390908; doi:10.1038/s41467-024-51779-8)
Supplement: Supplementary file 3 — Reporting Summary [file 41467_2024_51779_MOESM3_ESM.pdf]

nature portfolio

Corresponding author(s): DBPR NNCOMMS-23-44661C-Z

Last updated by author(s): 2024/08/13

Reporting Summary

Nature Portfolio wishes to improve the reproducibility of the work that we publish. This form provides structure for consistency and transparency in reporting. For further information on Nature Portfolio policies, see our Editorial Policies and the Editorial Policy Checklist.

Statistics

For all statistical analyses, confirm that the following items are present in the figure legend, table legend, main text, or Methods section.

n/a	Confirmed	
☐	☒	The exact sample size (*n*) for each experimental group/condition, given as a discrete number and unit of measurement
☐	☒	A statement on whether measurements were taken from distinct samples or whether the same sample was measured repeatedly
☐	☒	The statistical test(s) used AND whether they are one- or two-sided<br>*Only common tests should be described solely by name; describe more complex techniques in the Methods section.*
☒	☐	A description of all covariates tested
☐	☒	A description of any assumptions or corrections, such as tests of normality and adjustment for multiple comparisons
☐	☒	A full description of the statistical parameters including central tendency (e.g. means) or other basic estimates (e.g. regression coefficient) AND variation (e.g. standard deviation) or associated estimates of uncertainty (e.g. confidence intervals)
☐	☒	For null hypothesis testing, the test statistic (e.g. *F*, *t*, *r*) with confidence intervals, effect sizes, degrees of freedom and *P* value noted<br>*Give P values as exact values whenever suitable.*
☒	☐	For Bayesian analysis, information on the choice of priors and Markov chain Monte Carlo settings
☒	☐	For hierarchical and complex designs, identification of the appropriate level for tests and full reporting of outcomes
☒	☐	Estimates of effect sizes (e.g. Cohen's *d*, Pearson's *r*), indicating how they were calculated

Our web collection on statistics for biologists contains articles on many of the points above.

Software and code

Policy information about availability of computer code

Data collection	Data collection was performed using a human computer interface developed using Python 3.8 and PyCharm Community Edition 2021.2.2. The scripts for data collection can be provided upon reasonable request to the corresponding author.
Data analysis	Data analysis was performed using Matlab R2021b. The correlation analysis between duty cycle, pipe length, and max frequency was done in Microsoft Excel Version 2307 using the Regression tool in the Data Analysis toolbox.

For manuscripts utilizing custom algorithms or software that are central to the research but not yet described in published literature, software must be made available to editors and reviewers. We strongly encourage code deposition in a community repository (e.g. GitHub). See the Nature Portfolio guidelines for submitting code & software for further information.

Data

Policy information about availability of data

All manuscripts must include a data availability statement. This statement should provide the following information, where applicable:

- Accession codes, unique identifiers, or web links for publicly available datasets
- A description of any restrictions on data availability
- For clinical datasets or third party data, please ensure that the statement adheres to our policy

All data will be available in the University College London Research Data Repository before publication.

Research involving human participants, their data, or biological material

Policy information about studies with human participants or human data. See also policy information about sex, gender (identity/presentation), and sexual orientation and race, ethnicity and racism.

Reporting on sex and gender	Sex (biological attribute) was not considered in this study because as mentioned by one of the reviewers, the sample size was small and the variation observed may not me due to sex but due to other variables. Therefore, sex is mentioned as part of Future work. Sex was self-reported through the questionnaire filled by the participants during the studies. Consent was obtained to publish the outcomes of the studies.
Reporting on race, ethnicity, or other socially relevant groupings	Sex was not considered in the study as part of the main analysis but as part of Future Work. Sex was self-reported
Population characteristics	Participants did not suffer from any sensory or motor impairment (e.g. Parkinson's), and with healthy hand skin and no amputations. For the haptic feedback system evaluation, participants were 11 males and 7 females between 21 and 39 years old. For the handheld devices study, participants were 5 males and 5 females between 20 and 37 years old
Recruitment	Recruitment email with brief information of the study was sent to UCL's community.
Ethics oversight	University College London's Research Ethics Committee

Note that full information on the approval of the study protocol must also be provided in the manuscript.

Field-specific reporting

Please select the one below that is the best fit for your research. If you are not sure, read the appropriate sections before making your selection.

☒ Life sciences ☐ Behavioural & social sciences ☐ Ecological, evolutionary & environmental sciences

For a reference copy of the document with all sections, see nature.com/documents/nr-reporting-summary-flat.pdf

Life sciences study design

All studies must disclose on these points even when the disclosure is negative.

Sample size	Power analysis was conducted to identify that the minimum sample size for the evaluation of the haptic feedback system was three. For the calculation, we used handheld devices' experiment data (sensitivity standard deviation of 1.1 mN and stimuli differentiation across fingers standard deviation of 0.83 mm) with a power of 0.8 and a significance level of 0.01.
Data exclusions	No exclusions were made
Replication	Data is available at the UCL research data repository (DOI:10.5522/04/26169838), all the information of the components of the system and methods have been provided in the manuscripts. If further information is required, it can be provided by the corresponding author reasonable request.
Randomization	The order of the data collection was randomized across areas. The data collection order across fingers was ring, middle, index and thumb.
Blinding	Blinding was not relevant to the study.

Reporting for specific materials, systems and methods

We require information from authors about some types of materials, experimental systems and methods used in many studies. Here, indicate whether each material, system or method listed is relevant to your study. If you are not sure if a list item applies to your research, read the appropriate section before selecting a response.

Materials & experimental systems

n/a	Involved in the study
☒	☐ Antibodies
☒	☐ Eukaryotic cell lines
☒	☐ Palaeontology and archaeology
☒	☐ Animals and other organisms
☒	☐ Clinical data
☒	☐ Dual use research of concern
☒	☐ Plants

Methods

n/a	Involved in the study
☒	☐ ChIP-seq
☒	☐ Flow cytometry
☒	☐ MRI-based neuroimaging
